# Gallbladder disease and use of transdermal versus oral hormone replacement therapy in postmenopausal women: prospective cohort study

**DOI:** 10.1136/bmj.a386

**Published:** 2008-07-31

**Authors:** Bette Liu, Valerie Beral, Angela Balkwill, Jane Green, Siân Sweetland, Gillian Reeves

**Affiliations:** 1 Epidemiology Unit, University of Oxford, Oxford OX3 7LF

## Abstract

**Objective** To determine whether transdermal compared with oral use of hormone replacement therapy reduces the risk of gallbladder disease in postmenopausal women.

**Design** Prospective cohort study (Million Women Study).

**Setting** Women registered with the National Health Service (NHS) in England and Scotland.

**Participants** 1 001 391 postmenopausal women (mean age 56) recruited between 1996 and 2001 from NHS breast screening centres and followed by record linkage to routinely collected NHS hospital admission data for gallbladder disease.

**Main outcome measures** Adjusted relative risk and standardised incidence rates of hospital admission for gallbladder disease or cholecystectomy according to use of hormone replacement therapy.

**Results** During follow-up 19 889 women were admitted for gallbladder disease; 17 190 (86%) had a cholecystectomy. Compared with never users of hormone replacement therapy, current users were more likely to be admitted for gallbladder disease (relative risk 1.64, 95% confidence interval 1.58 to 1.69) but risks were substantially lower with transdermal therapy than with oral therapy (relative risk 1.17, 1.10 to 1.24 *v* 1.74, 1.68 to 1.80; heterogeneity P<0.001). Among women using oral therapy, equine oestrogens were associated with a slightly greater risk of gallbladder disease than estradiol (relative risk 1.79, 1.72 to 1.87 *v* 1.62, 1.54 to 1.70; heterogeneity P<0.001) and higher doses of oestrogen increased the risk more than lower doses: for equine oestrogens >0.625 mg, 1.91 (1.78 to 2.04) *v* ≤0.625 mg, 1.76 (1.68 to 1.84); heterogeneity P=0.02; estradiol >1 mg, 1.68 (1.59 to 1.77) *v* ≤1 mg, 1.44 (1.31 to 1.59); heterogeneity P=0.003. The risk of gallbladder disease decreased with time since stopping therapy (trend P=0.004). Results were similar taking cholecystectomy as the outcome. Standardised hospital admission rates per 100 women over five years for cholecystectomy were 1.1 in never users, 1.3 with transdermal therapy, and 2.0 with oral therapy.

**Conclusion** Gallbladder disease is common in postmenopausal women and use of hormone replacement therapy increases the risk. Use of transdermal therapy rather than oral therapy over a five year period could avoid one cholecystectomy in every 140 users.

## Introduction

Randomised controlled trials and observational studies have shown a clear increase in the risk of gallbladder disease (cholelithiasis, cholecystitis, or cholecystectomy as outcomes) with use of hormone replacement therapy by postmenopausal women.^1^ ^2^ ^3^ ^4^ Oestrogen administered orally is metabolised by the liver before entering the systemic circulation (“first pass metabolism”). Oestrogen administered transdermally avoids this first pass metabolism and it has been suggested that it might have a lesser effect on gallbladder disease than orally administered oestrogen.^1^ Hence we examined the relation between method of administration and type of hormone replacement therapy and the incidence of gallbladder disease in a large cohort of postmenopausal women in the United Kingdom.

## Methods

The Million Women Study is a population based prospective study that recruited 1.3 million women, aged 50-69 (mean age 56), from National Health Service (NHS) breast screening clinics in England and Scotland during 1996-2001. At recruitment the women provided information on their use of hormone replacement therapy, sociodemographic and anthropometric factors, and medical and reproductive history. Additional information, including repeat questions on hormone replacement therapy and questions on diet, was obtained at resurvey about three years after recruitment. Study questionnaires can be viewed at www.millionwomenstudy.org.uk. Participants provided written consent for inclusion and follow-up.

All participants were registered with the NHS at recruitment and their NHS number is a unique personal identifier used in all NHS health records, including the NHS breast cancer screening programme and admissions to NHS hospitals. Study participants were followed by record linkage using their unique NHS number and other personal details for deaths,^5^ cancer registrations,^5^ emigration,^5^ and hospital admissions,^6^ ^7^ thus providing close to complete follow-up for all these events. The hospital admission data include details of all NHS funded hospital admissions, including day surgery, dating from 1981 in Scotland^6^ and from 1997 in England.^7^ For every hospital record the primary reason for admission and up to 13 additional clinical diagnoses are coded using the *International Classification of Diseases* 10th revision (ICD-10)^8^; as well, up to 12 procedures are coded using the *Office of Population Censuses and Surveys Classification of Surgical Operations and Procedures*, fourth revision (OPCS 4).^9^ For these analyses the main outcome, gallbladder disease, is defined as the first hospital admission after recruitment to the study with a primary diagnosis of cholelithiasis or cholecystitis (K80 and K81, ICD-10) or a cholecystectomy (codes J181-J189, OPCS 4). Analyses were also done using as an outcome the first admission for cholecystectomy alone (codes J181-J189).

### Statistical analysis

We excluded women from analyses if they were premenopausal or perimenopausal (as previously defined^10^), had a history of cancer at baseline (except non-melanoma skin cancers; code C44, ICD-10), had a record of a hospital admission for gallbladder disease before recruitment, or whose hormone replacement therapy use was unknown. Person years were calculated from the date of recruitment to the date of first hospital admission for gallbladder disease, date of death, date of emigration, or the end of follow-up, whichever came first. For women recruited in Scotland the last date of follow-up was 31 December 2003 and for those in England the last date was 31 March 2005, corresponding to the dates that the hospital records were complete at the time of record linkage. We calculated person years from 1 April 1997 for the small proportion of women (5%) recruited in England before that date, as hospital admission data were not available before then. Sensitivity analyses were done by recalculating person years as in the main analysis but censoring women who had breast cancer (C50, ICD-10), cardiovascular disease (I20-I25, I60-I69, ICD-10), or thromboembolic disease (I26, I80-I82, ICD-10) at the date of diagnosis or admission to hospital for any of these events and also by censoring follow-up from 1 July 2002. In the sensitivity analyses we also excluded women recruited in England before the date that hospital admissions were available (5%) and those who reported pre-existing heart disease, stroke, or thrombosis at recruitment.

We used Cox regression to estimate the relative risk of hospital admission for gallbladder disease in relation to use of hormone replacement therapy. Analyses were routinely adjusted for area of recruitment (10 geographical regions), socioeconomic status in thirds (as defined previously^10^), body mass index (<22.5, 22.5-24.9, 25-27.4, 27.5-29.9, 30-34.9, ≥35 kg/m^2^), and parity (nulliparous, parous), and routinely stratified for age (two year age categories) and for hysterectomy (no, yes), as hysterectomy is known to alter the type of hormone replacement therapy prescribed. We also examined the effect of adjusting for other potential confounders including smoking,^11^ alcohol intake,^12^ physical activity,^13^ dietary factors (intake of fruit, vegetables, nuts, and coffee),^14^ ^15^ previous oral contraceptive use,^16^ treatment for hypertension,^4^ reporting of high cholesterol levels, and a history of stroke, heart disease, thrombosis, or bilateral oophorectomy.

We categorised participants according to their reported use of hormone replacement therapy at recruitment. Proprietary hormone replacement therapy preparations were classified on the basis of method of administration and hormonal constituents using the *British National Formulary*.^17^ We defined women as users of oral therapy if they were taking any tablet formulation of hormone replacement therapy or users of transdermal therapy if they were using a patch or gel formulation of oestrogen with or without a progestogen (patch or tablet form). Women using oestrogen implants were categorised separately. When the type of hormone replacement therapy could not be categorised, we included women in the “other and unknown” category and included them in all analyses. For past users of hormone replacement therapy we assumed the time since last use increased by one year for each year of follow-up. Furthermore, as hormone use may have changed during follow-up, we examined the potential effect of this by using information on use of hormone replacement therapy reported at resurvey.

We calculated standardised incidence rates for hospital admissions for cholecystectomy in users of hormone replacement therapy, taking incidence rates in never users as the standard and adjusting for age, hysterectomy, region, socioeconomic group, body mass index, and parity. Relative risks are reported with 95% confidence intervals. Analyses were done using Stata 9.2 statistical software.

## Results

Overall, 1 001 391 women were included in the analyses, representing 95% of all women in the cohort who were postmenopausal at recruitment. In total, 32% were current users of hormone replacement therapy and 18% were past users. Among current users, 77% used an oral formulation, 18% a transdermal formulation, and 5% other or unknown types. Table 1 shows the baseline characteristics of participants by use of hormone replacement therapy. Past and current users differed primarily from never users as they were more likely to have used oral contraceptives and to have had a hysterectomy or bilateral oophorectomy. Current users of transdermal and oral formulations were similar for most characteristics except that hysterectomy and bilateral oophorectomy were more common in women using transdermal therapy. On the basis of resurvey data collected a mean of 2.8 years after recruitment from 65% of participants, few women who were never users or past users at recruitment became current users subsequently (1% and 3% per year, respectively). The proportion of current users who stopped use after recruitment was similar for users of oral therapy and transdermal therapy (10% and 9% per year, respectively). Among current users at recruitment who were still current users at resurvey, 1% per year who initially used oral therapy had changed to transdermal therapy, whereas 4% per year who used transdermal therapy had changed to oral therapy.

**Table 1 tbl1:** Characteristics of participants and follow-up for gallbladder disease according to hormone replacement therapy use at recruitment. Values are percentages (number) unless stated otherwise

Characteristic	Never users	Past users	Method of administration in current users		
			Oral	Transdermal	Other or unknown*
No of women	478 180	181 857	263 871	60 247	17 236
Mean (SD) age (years)	58.2 (4.4)	57.5 (4.0)	56.5 (3.9)	56.4 (3.9)	56.1 (4.1)
Mean (SD) body mass index (kg/m^2^)	26.5 (4.9)	26.5 (4.6)	25.6 (4.3)	26.1 (4.3)	26.0 (4.4)
Upper third socioeconomic group	31.6 (149 877)	33.4 (60 195)	34.8 (91 035)	36.0 (21 490)	34.8 (5938)
Current smoker	20.2 (90 383)	20.9 (35 804)	20.8 (51 891)	19.0 (10 813)	20.2 (3249)
Mean (SD) alcohol intake (g/day)	5.3 (7.1)	6.2 (7.5)	6.8 (7.8)	6.8 (7.8)	6.6 (7.8)
Mean (SD) parity	2.2 (1.3)	2.2 (1.2)	2.1 (1.2)	2.2 (1.2)	2.2 (1.2)
Ever used oral contraceptives	46.3 (219 306)	60.8 (109 371)	64.4 (168 154)	61.8 (36 775)	65.4 (11 123)
Treated for hypertension	18.0 (85 834)	17.0 (30 930)	17.0 (44 762)	17.5 (10 506)	16.4 (2826)
Reporting high cholesterol level	12.7 (60 691)	13.7 (24 937)	11.0 (28 981)	14.1 (8465)	10.7 (1845)
Heart disease, stroke, or thrombosis	11.0 (52 416)	11.3 (20 513)	8.2 (21 416)	10.9 (6540)	9.8 (1696)
Hysterectomy	14.8 (70 213)	27.1 (48 875)	32.6 (85 521)	73.5 (44 168)	65.9 (11 301)
Bilateral oophorectomy	3.1 (14 024)	10.0 (17 286)	15.2 (38 040)	36.8 (20 613)	44.9 (7292)
Person years of follow-up	2 926 227	1 095 950	1 609 704	365 590	105 340
No admitted with gallbladder disease	7581	3708	6914	1249	437

Percentages calculated excluding women with missing values.

*Includes women using oestrogen implants.

Participants were followed for 6 102 811 person years (mean 6.1, interquartile range 5.4-7.0 years per woman). During follow-up 19 889 women had a first admission for gallbladder disease, 17 190 (86%) of whom had a cholecystectomy. Admissions for gallbladder disease occurred a mean of 3.3 years after recruitment.

Figure 1 shows the relation between the risk of gallbladder disease and use of hormone replacement therapy. Compared with never users, current and past users had an increased risk of gallbladder disease, although the risk was significantly greater in current users (relative risk 1.64, 95% confidence interval 1.58 to 1.69) than in past users (1.27, 1.22 to 1.32; test for heterogeneity χ^2^_1_=158, P<0.001). Among past users the risk of gallbladder disease declined with increasing time since last use (P=0.004 for linear trend); for women who stopped hormone use more than 10 years previously, however, the risk remained significantly greater than in never users (1.19, 1.10 to 1.29). Results were similar when cholecystectomy was taken as the outcome: on the basis of 6406 cholecystectomies in never users, 7570 in current users, and 3214 in past users, the relative risk for current users was 1.69 (1.63 to 1.75) and for past users was 1.29 (1.23 to 1.34).

**Figure fig1:**
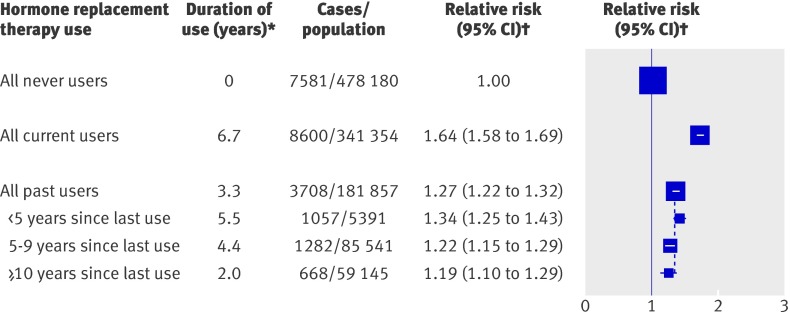
**Fig 1** Relative risk of hospital admission for gallbladder disease by hormone replacement therapy use at recruitment. Dotted line represents overall relative risk for past users compared with never users. *Mean years of use estimated for cases at time of admission in current users and at recruitment for past users, using resurvey information. †Relative risk compared to never users stratified by age and hysterectomy and adjusted for region, socioeconomic group, body mass index, and parity. Numbers for categories of time since last use do not sum to total for past users owing to missing values

Figure 2 shows results by the method of hormone administration. Current users of oral, transdermal, and implanted therapies had higher risks of gallbladder disease than never users but the risks were significantly different depending on the method of administration (heterogeneity χ^2^_3_=170, P<0.001). Transdermal therapy conferred a substantially lower risk of gallbladder disease than oral therapy (relative risk 1.17, 1.10 to 1.24 *v* 1.74, 1.68 to 1.80; heterogeneity χ^2^_1_=166, P<0.001). The lower risk with transdermal therapy compared with oral therapy was also seen when cholecystectomy alone was used as the outcome: relative risk 1.18 (1.10 to 1.27) and 1.80 (1.74 to 1.87), respectively.

**Figure fig2:**
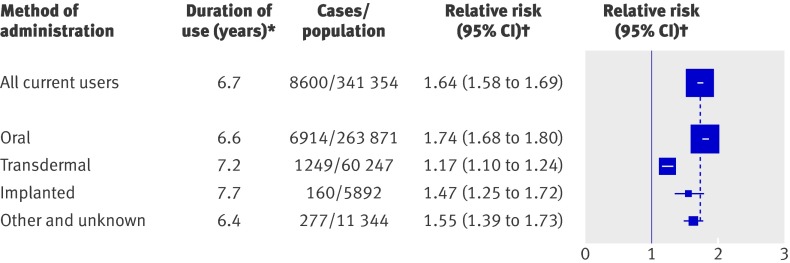
**Fig 2** Relative risk of hospital admission for gallbladder disease in current users of hormone replacement therapy compared with never users by method of administration. Dotted line represents overall relative risk for all current users compared with never users. *Mean years of use estimated for cases at time of admission for gallbladder disease, using resurvey information. †Relative risk compared with never users stratified by age and hysterectomy and adjusted for region, socioeconomic group, body mass index, and parity

Relative risks did not change substantially when simultaneous adjustment was made for the additional potential confounders of smoking status, alcohol intake, physical activity, previous oral contraceptive use, treatment for hypertension, reporting of high cholesterol levels, dietary factors, and history of stroke, heart disease, thrombosis, or bilateral oophorectomy: for transdermal therapy the relative risk changed from 1.17 to 1.21 and for oral therapy from 1.74 to 1.79.

The relative risk of gallbladder disease in current users of oral and transdermal therapies was also compared with never users in various subgroups of women that had been defined a priori (fig 3).^10^ ^18^ ^19^ The relative risks differed significantly only by body mass index, decreasing with increasing body mass index: relative risk was 1.88 (1.77 to 2.01) for a body mass index <25 kg/m^2^ and 1.47 (1.30 to 1.67) for a body mass index ≥35 kg/m^2^; P=0.001 for trend.

**Figure fig3:**
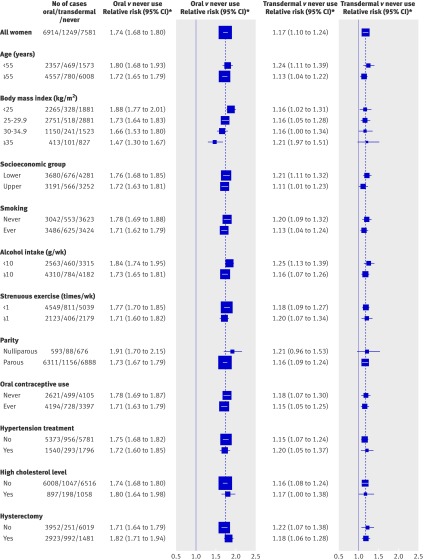
**Fig 3** Relative risk of hospital admission for gallbladder disease in current users of oral and transdermal hormone replacement therapy compared with never users in various subgroups. Dotted lines represent overall relative risk for all users of oral therapy compared with never users and all users of transdermal therapy compared with never users, respectively. *Relative risk compared with never users stratified by age and hysterectomy and, when appropriate, adjusted for region, socioeconomic group, body mass index, and parity

Figure 4 shows results for various features of the oral hormone replacement therapies. The risk of gallbladder disease was not significantly related to duration of use but did vary significantly by type of oestrogen and dose (fig 4). Women using equine oestrogens had a significantly higher risk of gallbladder disease than those using estradiol: relative risk 1.79 (1.72 to 1.87) *v* 1.62 (1.54 to 1.70); heterogeneity χ^2^_1_=13.7, P<0.001. Furthermore, use of both equine oestrogen and estradiol at higher doses were associated with greater risks than lower doses: with equine oestrogen the relative risks were 1.76 (1.68 to 1.84) for ≤0.625 mg and 1.91 (1.78 to 2.04) for >0.625 mg (heterogeneity χ^2^_1_=5.2, P=0.02); with estradiol the risks were 1.44 (1.31 to 1.59) for ≤1 mg and 1.68 (1.59 to 1.77) for >1 mg (heterogeneity χ^2^_1_=8.6, P=0.003). No substantial variation was found in risk between users of oestrogen only, oestrogen plus progestogen, tibolone, and other types (heterogeneity χ^2^_3_=7.1, P=0.07). Within each category of oestrogen type and dose, the type of progestogen used and frequency of administration varied considerably, limiting comparisons (table 2). Sequential norgestrel is represented in each category and seems to be associated with a lower risk of gallbladder disease than the other oral preparations; the lack of information on the other progestogens, however, makes these findings difficult to interpret.

**Figure fig4:**
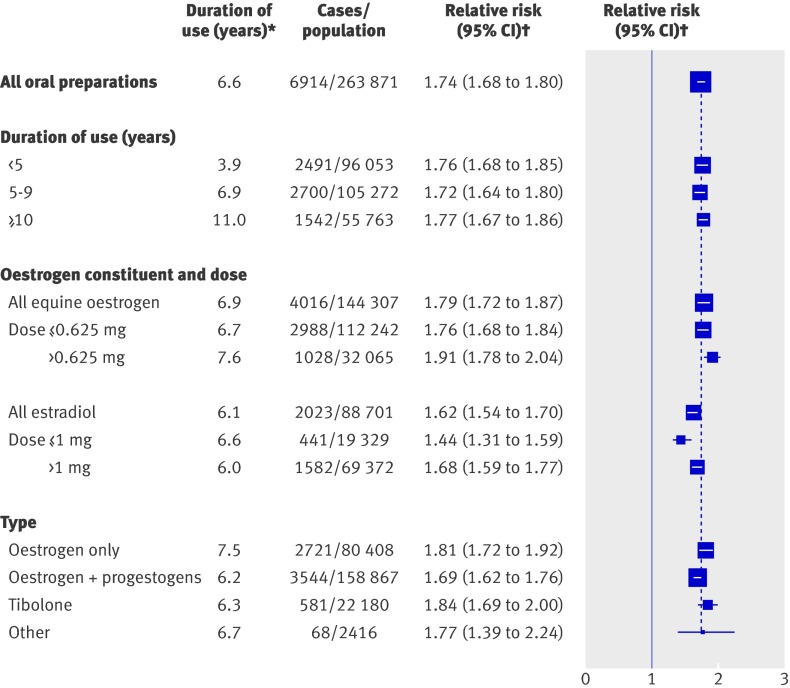
**Fig 4** Relative risk of hospital admission for gallbladder disease in current users of oral hormone replacement therapy compared with never users by duration of use and type of therapy reported at recruitment. Dotted line represents overall relative risk for all users of oral therapy compared with never users. *Mean years of use estimated for cases at time of admission for gallbladder disease, using resurvey information. †Relative risk compared with never users stratified by age and hysterectomy and adjusted for region, socioeconomic group, body mass index, and parity. Numbers in subcategories do not necessarily sum to totals owing to missing values

**Table 2 tbl2:** Relative risk of hospital admission for gallbladder disease in current users of oral hormone replacement therapy compared with never users by type of oestrogen, dose, and addition of progestogens*

Type and dose of oestrogen	Progestogen use	No of women with gallbladder disease	Relative risk (95%CI)
Equine oestrogen (mg):			
≤0.625	None	1250	1.76 (1.64 to 1.87)
	Norgestrel (sequential†)	981	1.69 (1.58 to 1.80)
	Medroxyprogesterone (continuous‡)	674	1.92 (1.77 to 2.08)
>0.625	None	704	2.09 (1.93 to 2.28)
	Norgestrel (sequential†)	311	1.66 (1.48 to 1.86)
Estradiol (mg):			
≤1	None	253	1.55 (1.37 to 1.77)
	Norgestrel (sequential†)	70	1.33 (1.05 to 1.69)
>1	None	275	1.79 (1.58 to 2.03)
	Norgestrel (sequential†)	169	1.34 (1.15 to 1.56)
	Norethisterone (sequential†)	305	1.54 (1.37 to 1.73)
	Norethisterone (continuous‡)	755	1.80 (1.67 to 1.95)

*Progestogen types are listed where there were more than 50 women with gallbladder disease.

†Progestogens taken for 10-14 days of each monthly cycle for most women.

‡Progestogens taken daily.

Figure 5 shows results for various features of the transdermal preparations. Estradiol was the only type of oestrogen administered transdermally and no significant differences were found by duration of its use, dose, or whether it was used alone or with a progestogen (fig 5). Progestogens administered orally or transdermally did not appreciably alter the relative risk associated with use of transdermal oestrogen.

**Figure fig5:**
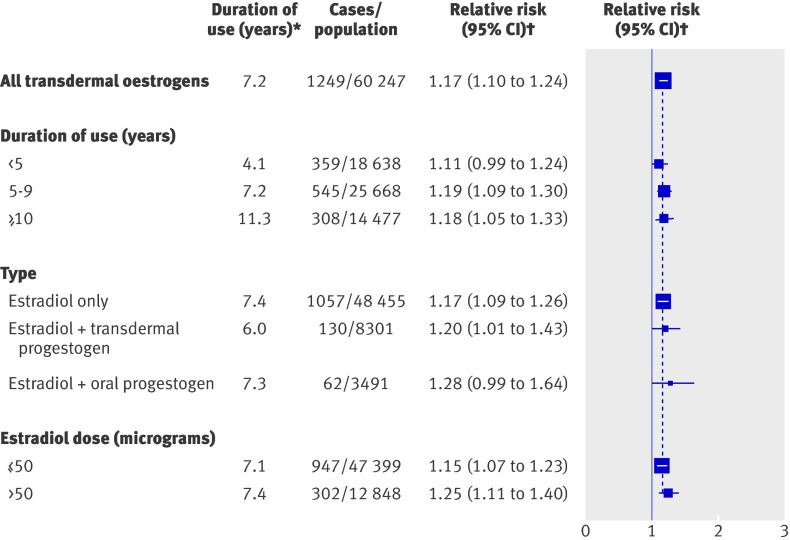
**Fig 5** Relative risk of hospital admission for gallbladder disease in current users of transdermal hormone replacement therapy compared with never users by duration of use and type of therapy reported at recruitment. Dotted line represents overall relative risk for all users of transdermal therapy compared with never users. *Mean years of use estimated for cases at time of admission for gallbladder disease, using resurvey information. †Relative risk compared with never users stratified by age and hysterectomy and adjusted for region, socioeconomic group, body mass index, and parity. Numbers in subcategories do not necessarily sum to totals owing to missing values

Table 3 shows the main findings for hormone replacement therapy and gallbladder disease compared with results from the sensitivity analyses where follow-up was censored and additional exclusions were applied to the cohort. Although the relative risk estimates were marginally higher than in the main analysis the relation between never users, current users, and past users of hormone replacement therapy did not change appreciably. Also, transdermal therapy consistently conferred a lower risk of gallbladder disease than oral therapy.

**Table 3 tbl3:** Sensitivity analyses of risk of hospital admission for gallbladder disease according to hormone replacement therapy (HRT) use. Values are relative risks (95% confidence intervals) unless stated otherwise

	Main model	Follow-up censored at diagnosis or admission for medical illness*	Follow-up censored at diagnosis or admission for medical illness* or on July 2002
No of women	1 001 391	857 503	857 503
Mean person years of follow-up	6.1	5.8	3.4
No admitted to hospital for gallbladder disease	19 889	15 248	8330
HRT use compared with never users:	—	—	—
All current users	1.64 (1.58 to 1.69)	1.69 (1.62 to 1.75)	1.82 (1.72 to 1.91)
All past users	1.27 (1.22 to 1.32)	1.31 (1.25 to 1.37)	1.34 (1.26 to 1.43)
Current users of oral HRT	1.74 (1.68 to 1.80)	1.79 (1.72 to 1.86)	1.94 (1.84 to 2.05)
Current users of transdermal HRT	1.17 (1.10 to 1.24)	1.21 (1.12 to 1.30)	1.20 (1.09 to 1.32)

*In all sensitivity analyses women who were recruited in England before hospital admission data were available and those who reported pre-existing heart disease, stroke, or thrombosis were excluded. Follow-up was censored if the participant had breast cancer, cardiovascular disease, or thromboembolic disease at date of diagnosis or admission to hospital.

The standardised incidence rates per 100 women over five years in never users of hormone replacement therapy were 1.3 (95% confidence interval 1.3 to 1.3) for hospital admissions with gallbladder disease and 1.1 (1.1 to 1.1) for cholecystectomy alone. The corresponding rates for current users of transdermal therapy were 1.5 (1.4 to 1.6) and 1.3 (1.2 to 1.4) and for current users of oral therapy were 2.3 (2.2 to 2.3) and 2.0 (1.9 to 2.0). From these figures over five years the absolute difference in risk between current users of oral therapy and transdermal therapy was 0.8 per 100 women for gallbladder disease and 0.7 per 100 women for cholecystectomy. Hence among women using transdermal therapy compared with oral therapy over five years, one fewer admission for gallbladder disease would be expected in every 125 women and one fewer admission for cholecystectomy in every 140 women.

## Discussion

Use of hormone replacement therapy by postmenopausal women increases the risk of gallbladder disease. This finding agrees with previous reports.^1^ ^2^ ^3^ ^4^ We also found a substantially lower risk of gallbladder disease with use of transdermal therapy than with oral therapy (relative risk 1.17 *v* 1.74). Oestrogens administered orally are absorbed in the gut and travel through the portal vein to the liver where they are extensively metabolised through conjugation before entering the systemic circulation. The metabolites are excreted through the bile and urine.^20^ Transdermal oestrogens are administered in lower doses than oral oestrogens and are absorbed through the skin and directly into the circulation. By avoiding first pass metabolism there should be lower concentrations of oestrogens and their metabolites in the bile and this may well explain the lower risk of gallbladder disease that we observed with transdermal therapy.

Among users of oral hormone therapy we found a dose effect, with increased relative risks of gallbladder disease among users of higher doses compared with lower doses. These findings suggest that the concentration of oestrogen and its metabolites in the bile affect the risk of gallbladder disease. Use of equine oestrogen was associated with a slightly higher risk of gallbladder disease than estradiol and this may be because the equine preparations consist largely of conjugated oestrogens, which are metabolised somewhat differently from estradiol^20^; however, it is unknown what effects this may have on biliary concentrations of hormone. Oestrogen implants, which can result in high blood concentrations of oestrogen^20^ ^21^ ^22^ but avoid the first pass metabolism, were also found to increase the relative risk of gallbladder disease to a level somewhere between oral users and transdermal users.

Our results suggest that the addition of progestogens to oestrogen therapy does not have a large additional effect on the risk of gallbladder disease. This is in agreement with findings from the women’s health initiative randomised controlled trial.^1^ Furthermore, among users of transdermal oestrogens, the risks did not vary appreciably if the progestogens were administered orally or transdermally, although owing to the number of cases statistical power was limited.

In past users of hormone replacement therapy the risk of gallbladder disease was lower than in current users and decreased gradually with increasing time since therapy stopped although some excess risk still remained 10 years after stopping. This finding suggests that oestrogen induces some persistent change in the gallbladder. Similar findings have been described in other studies^3^ ^23^ and one possible explanation is the formation of asymptomatic gallstones, which continue to increase a woman’s risk of gallbladder disease long after stopping hormone replacement therapy.^23^ Results from one study suggest that the risk of gallbladder disease may increase with increasing duration of use^3^; however we found no significant trends. Finally, we did not compare the risks associated with past use of oral and transdermal formulations because a large proportion of past users provided no information on the type of hormone replacement therapy used and evidence suggests that recall of brand names by past users is poor.^24^

Among users of oral hormone replacement therapy the relative risk, but not the absolute risk, of gallbladder disease associated with hormone replacement therapy was found to decrease with increasing body mass index. A similar attenuation of the hormone replacement therapy associated relative risks in overweight and obese women has been observed with breast cancer^25^ and endometrial cancer.^18^ The absolute risk of gallbladder disease increases with increasing body mass index^26^ probably because circulating levels of endogenous oestradiol are higher in overweight and obese women and the addition of exogenous oestrogens may alter total oestrogen levels to a lesser extent than in thinner women (see fig 3). It has been suggested that the attenuated relative risks with increasing body mass index might be because we could not exclude every woman who had a cholecystectomy before recruitment, although additional analyses suggest this is unlikely.

### Strengths and limitations

Our results are based on hormone replacement therapy use reported at recruitment and although some changes in use occurred during follow-up this would not substantially alter our findings. Admissions for gallbladder disease occurred a mean of 3.3 years after recruitment and, on the basis of resurvey data, a small proportion of never users, only 1% annually, started use after recruitment. The proportion of current users stopping therapy was similar for women using oral and transdermal preparations and few women switched between oral and transdermal therapies. Sensitivity analyses showed that our estimates of relative risk were essentially unchanged after censoring women at major events that may alter the use of hormone replacement therapy. Also, after censoring follow-up at July 2002 (to coincide with publication of the first results from the women’s health initiative trials^27^), significant differences between the effect of transdermal therapy and oral therapy persisted.

In this cohort, the findings relating to the method of administration of hormone replacement therapy are specific for gallbladder disease, as we found no differences between transdermal therapy and oral therapy for increased risk of breast cancer^10^ and ovarian cancer,^19^ or the reduced risk of fracture.^28^ Prescribing of transdermal oestrogen rather than oral oestrogen differs according to some factors such as history of hysterectomy and bilateral oophorectomy (see table 1) but we were able to adjust for these as well as other potential confounders that could influence the choice of hormone replacement therapy such as socioeconomic status, body mass index, and other medical history. 

Although some non-NHS funded admissions would not be included in our follow-up, privately funded treatment in the United Kingdom is limited,^29^ and we have previously shown that in this cohort such admissions are uncommon, as most self reported cholecystectomies were included in our linked NHS hospital admission data.^30^ We excluded from the analyses women with a record of admission for gallbladder disease before recruitment as most of these women will have had a cholecystectomy and would no longer be at risk of an event. For women recruited in Scotland, hospital records were available from 1981 (when participants were a mean age of 36). In these women, had we not excluded those with an admission before recruitment, the relative risks for gallbladder disease in current users of hormone therapy compared with never users would have been 1.70 instead of 1.71, and the relative risks for transdermal therapy versus oral therapy would have been 1.13 versus 1.83 instead of 1.13 versus 1.85. For women recruited in England, hospital records were available only from 1997; however, the small difference in relative risks in Scotland suggests that not excluding comparable women in England would have a minimal effect on our estimates.

Despite these limitations, the study has several strengths. All women were registered with the NHS, and by linking to NHS hospital records we had virtually complete follow-up, with objective recording of gallbladder disease. The large sample size allowed us to compare reliably a variety of types of hormone replacement therapy. The prospective design ensured that use of hormone replacement therapy was ascertained before outcomes thereby reducing biases resulting from recall or differential prescribing, and we were able to adjust for many potential confounders and found our results to be consistent. Reporting of hormone replacement therapy use in this population compares well with prescription data,^31^ and outcomes were coded by the NHS hospitals, independent of the study investigators and participants. Additionally, both the accuracy of the linkage process and the coding of gallbladder disease has been shown to be good^6^ ^30^ and as misclassification of hormone replacement therapy use at recruitment or of gallbladder disease from the hospital records should be non-differential, this would result in an underestimate of the effects found.

### Implications for practice

Gallbladder disease is common in middle aged women. In the UK over five years an estimated 1.1% of middle aged women who have never used hormone replacement therapy are admitted to hospital for a cholecystectomy. Use of transdermal oestrogen increases the risk to 1.3% and use of oral oestrogens increases this to 2.0%. Transdermal hormone replacement therapy is, however, generally more costly than oral therapy and can cause local skin reactions.^20^ ^21^ Whereas the number of women using hormone replacement therapy halved between 2002 and 2005, about 1 million UK women were taking it in 2005 and most used oral preparations.^32^ For women who choose to use hormone replacement therapy, one cholecystectomy could be avoided for every 140 users of transdermal therapy rather than oral therapy over a five year period.

**What is already known on this topic**

Hormone replacement therapy increases the risk of gallbladder disease in postmenopausal women
Oestrogens given transdermally rather than orally avoid first pass metabolism by the liver and may have a lesser effect on the risk of gallbladder disease than oral oestrogens

**What this study adds**

Use of transdermal oestrogens are associated with a substantially lower risk of gallbladder disease than use of oral oestrogens
Over five years one cholecystectomy could be avoided for every 140 postmenopausal women who use transdermal rather than oral hormone replacement therapy

## Notes

We thank the women who participated in the Million Women Study, the study steering committee, coordinating centre staff, and collaborating NHS breast screening centres. We also thank ISD Scotland, the Information Centre for Health and Social Care, and Northgate Solutions for the linkage of hospital records.

Ethical approval: The study has been approved by the Eastern multicentre research ethics committee.

Provenance and peer review: Not commissioned; externally peer reviewed.

## Notes

**Cite this as:** *BMJ* 2008;337:a386

## References

[1] Cirillo DJ, Wallace RB, Rodabough RJ, Greenland P, LaCroix AZ, Limacher MC, et al. Effect of estrogen therapy on gallbladder disease. JAMA 2005;293:330-9. 15657326 10.1001/jama.293.3.330

[2] Simon JA, Hunninghake DB, Agarwal SK, Lin F, Cauley JA, Ireland CC, et al. Effect of estrogen plus progestin on risk for biliary tract surgery in postmenopausal women with coronary artery disease. Ann Intern Med 2001;135:493-501. 11578152 10.7326/0003-4819-135-7-200110020-00008

[3] Grodstein F, Colditz GA, Stampfer MJ. Postmenopausal hormone use and cholecystectomy in a large prospective study. Obstet Gynecol 1994;83:5-11. 8272307

[4] Mamdani MM, Tu K, van Walraven C, Austin PC, Naylor DC. Postmenopausal estrogen replacement therapy and increased rates of cholecystectomy and appendectomy. CMAJ 2000;162:1421-4. 10834045 PMC1232454

[5] Million Women Study Collaborators. The million women study: design and characteristics of the study population. Breast Cancer Res 1999;1:73-80. 11056681 10.1186/bcr16 PMC13913

[6] Kendrick S, Clarke J. The Scottish record linkage system. Health Bull (Edinb) 1993;51:72-9. 8514493

[7] Information Centre. Hospital Episode Statistics. 2007. www.hesonline.nhs.uk.

[8] World Health Organization. International statistical classification of diseases and related health problems. 10th revision. Geneva: WHO, 1992.

[9] Office of population censuses and surveys. Classification of surgical operations and procedures. Fourth revision. London: Crown copyright, 2000.

[10] Million Women Study Collaborators. Breast cancer and hormone-replacement therapy in the million women study. Lancet 2003;362:419-27. 12927427 10.1016/s0140-6736(03)14065-2

[11] Murray F, Logan R, Hannaford P, Kay C. Cigarette smoking and parity as risk factors for the development of symptomatic gall bladder disease in women: results of the Royal College of General Practitioners’ oral contraception study. Gut 1994;35:107-11. 8307429 10.1136/gut.35.1.107 PMC1374643

[12] Leitzmann MF, Tsai C-J, Stampfer MJ, Rimm EB, Colditz GA, Willett WC, et al. Alcohol consumption in relation to risk of cholecystectomy in women. Am J Clin Nutr 2003;78:339-47. 12885719 10.1093/ajcn/78.2.339

[13] Leitzmann MF, Giovannucci EL, Rimm EB, Stampfer MJ, Spiegelman D, Wing AL, et al. The relation of physical activity to risk for symptomatic gallstone disease in men. Ann Intern Med 1998;128:417-25. 9499324 10.7326/0003-4819-128-6-199803150-00001

[14] Leitzmann MF, Stampfer MJ, Willett WC, Spiegelman D, Colditz GA, Giovannucci EL. Coffee intake is associated with lower risk of symptomatic gallstone disease in women. Gastroenterology 2002;123:1823-30. 12454839 10.1053/gast.2002.37054

[15] Tsai C-J, Leitzmann MF, Willett WC, Giovannucci EL. Fruit and vegetable consumption and risk of cholecystectomy in women. Am J Med 2006;119:760-7. 16945611 10.1016/j.amjmed.2006.02.040

[16] Thijs C, Knipschild P. Oral contraceptives and the risk of gallbladder disease: a meta-analysis. Am J Public Health 1993;83:1113-20. 8342719 10.2105/ajph.83.8.1113 PMC1695167

[17] Mehta D. British national formulary. 37th ed. London: BMA and Royal Pharmaceutical Society of Great Britain, 1999.

[18] Beral V, Bull D, Reeves G, Million Women Study Collaborators. Endometrial cancer and hormone-replacement therapy in the million women study.[see comment]. Lancet 2005;365:1543-51. 15866308 10.1016/S0140-6736(05)66455-0

[19] Million Women Study Collaborators. Ovarian cancer and hormone replacement therapy in the million women study. Lancet 2007;369:1703-10. 17512855 10.1016/S0140-6736(07)60534-0

[20] Sweetman S, ed. Martindale: the complete drug reference. 35th ed. London: Pharmaceutical Press, 2007.

[21] Lumsden M. Hormone replacement therapy: regimen and routes of administration. In: Critchley H, Gebbie A, Beral V, eds. Menopause and hormone replacement. London: RCOG Press, 2004:187-203.

[22] Gangar K, Cust M, Whitehead M. Symptoms of oestrogen deficiency associated with supraphysiological plasma oestradiol concentrations in women with oestradiol implants. BMJ 1989;299:601-2. 10.1136/bmj.299.6699.601-a PMC1837423 2508819

[23] Petitti DB, Sidney S, Perlman JA. Increased risk of cholecystectomy in users of supplemental estrogen. Gastroenterology 1988;94:91-5. 3335303 10.1016/0016-5085(88)90614-2

[24] Coulter A, Vessey M, McPherson K, Crossley B. The ability of women to recall their oral contraceptive histories. Contraception 1986;33:127-37. 3698594 10.1016/0010-7824(86)90079-x

[25] Collaborative group on hormonal factors in breast cancer. Breast cancer and hormone replacement therapy: collaborative reanalysis of data from 51 epidemiological studies of 52705 women with breast cancer and 108411 women without breast cancer. Lancet 1997;350:1047-59. 10213546

[26] Liu B, Balkwill A, Spencer E, Beral V. Relationship between body mass index and length of hospital stay for gallbladder disease. J Pub Health (Oxf) 2008;30:161-6. 18308742 10.1093/pubmed/fdn011

[27] Writing group for the Women’s Health Initiative Investigators. Risks and benefits of estrogen plus progestin in healthy postmenopausal women: principal results from the women’s health initiative randomized controlled trial. JAMA 2002;288:321-33. 12117397 10.1001/jama.288.3.321

[28] Banks E, Beral V, Reeves G, Balkwill A, Barnes I. Fracture incidence in relation to the pattern of use of hormone therapy in postmenopausal women. JAMA 2004;291:2212-20. 15138243 10.1001/jama.291.18.2212

[29] Williams B, Whatmough P, McGill J, Rushton L. Patients and procedures in short-stay independent hospitals in England and Wales, 1997-1998. J Public Health Med 2000;22:68-73. 10774907 10.1093/pubmed/22.1.68

[30] Liu B, Sweetland S, Beral V, Green J, Balkwill A, Casabonne D. Self-reported information on joint replacement and cholecystectomy agrees well with that in medical records. J Clin Epidemiol 2007;60:1190-4. 17938062 10.1016/j.jclinepi.2007.02.007

[31] Banks E, Beral V, Cameron R, Hogg A, Langley N, Barnes I, et al. Agreement between general practice prescription data and self-reported use of hormone replacement therapy and treatment for various illnesses. J Epidemiol Biostat 2001;6:357-63. 12036270 10.1080/13595220152601837

[32] Watson J, Wise L, Green J. Prescribing of hormone therapy for the menopause, tibolone, and bisphosphonates in women in the UK between 1991 and 2005. Eur J Clin Pharmacol 2007;63:843-9. 17598097 10.1007/s00228-007-0320-6

